# MicroRNA-506 suppresses tumor proliferation and metastasis in colon cancer by directly targeting the oncogene EZH2

**DOI:** 10.18632/oncotarget.5309

**Published:** 2015-10-03

**Authors:** Yi Zhang, Changwei Lin, Guoqing Liao, Sheng Liu, Jie Ding, Fang Tang, Zhenran Wang, Xingsi Liang, Bo Li, Yangchao Wei, Qi Huang, Xuan Li, Bo Tang

**Affiliations:** ^1^ Department of Gastrointestinal Surgery, Xiangya Hospital, Central South University, 410008, PR China; ^2^ Department of Oncological Surgery, Affiliated Hospital of Xuzhou Medical College, 221000, PR China; ^3^ Department of Gastrointestinal Surgery, Third Xiangya Hospital, Central South University, 410008, PR China; ^4^ Department of Gastrointestinal Surgery, Guizhou Provincial People's Hospital, 550000, PR China; ^5^ Department of Hepatobiliary Surgery, Affiliated Hospital of Guilin Medical University, 541000, PR China

**Keywords:** miR-506, EZH2, proliferation, metastasis, colon cancer

## Abstract

Increasing evidence reveals that aberrant expression of microRNA contributes to the development and progression of colon cancer, but the roles of microRNA-506 (miR-506) in colon cancer remain elusive. Here, we demonstrated that miR-506 was down-regulated in colon cancer tissue and cells and that miR-506 expression was inversely correlated with EZH2 expression, tumor size, lymph node invasion, TNM stage and metastasis. A high level of miR-506 identified patients with a favorable prognosis. *In vitro* and *in vivo* experiments confirmed that miR-506 inhibits the proliferation and metastasis of colon cancer, and a luciferase reporter assay confirmed that EZH2 is a direct and functional target of miR-506 via the 3′UTR of EZH2. The restoration of EZH2 expression partially reversed the proliferation and invasion of miR-506-overexpressing colon cancer cells. Moreover, we confirmed that the miR-506-EZH2 axis inhibits proliferation and metastasis by activating/suppressing specific downstream tumor-associated genes and the Wnt/β-catenin signaling pathway. Taking together, our study sheds light on the role of miR-506 as a suppressor for tumor growth and metastasis and raises the intriguing possibility that miR-506 may serve as a new potential marker for monitoring and treating colon cancer.

## INTRODUCTION

Colorectal cancer is the fourth most common form of cancer in humans. Each year, more than 600,000 die from colorectal cancer [1]. The majority of these deaths are caused by the progression of the tumor to metastatic disease. The 5-year survival rate for metastatic colorectal cancer is 10–15%, compared to 40%–90% for non-metastatic colorectal cancer. Thus, targeting proliferation and metastasis is an important strategy for treating colorectal cancer and improving patient outcomes.

MicroRNAs (miRNAs) are endogenous non-coding small RNA molecules that regulate the expression of target genes, typically via imperfect base-pairing to the 3′untranslated region (UTR) of a target mRNA, which leads to mRNA degradation or the inhibition of translation [2]. Recent studies have demonstrated that miRNAs can function as tumor suppressors or oncogenes and can play essential roles in the regulation of various cellular processes, including cell proliferation, differentiation, death and mobility [3]. Therefore, miRNA profiling is being utilized to identify potential tumor subtypes, diagnose cancers, determine treatment plans and predict patient outcomes [4, 5].

miR-506 is a component of an X chromosome-linked miRNA cluster. The expression pattern of miR-506 is complicated, even contradictory, in different types of cancer. miR-506 has been demonstrated to act as an oncogene in melanomas. In contrast, miR-506 functions as a tumor suppressor in ovarian [6] and cervical [7] cancer and suppresses malignant transformation in lung cancer [8]. These findings indicate the complex role of miR-506 in cancer development. Few reports have described the role of miR-506 in colon cancer. Tong JL [9] reported that miR-506 can confer chemoresistance in colon cancer, but the biological functions of miR-506 in colon cancer proliferation and metastasis are unclear and require further exploration.

Enhancer of zeste homolog 2 (EZH2) is a member of the Polycomb group (PcG) protein family. The EZH2 gene maps to chromosome 7q35 and contains 20 exons and 19 introns [10]. EZH2, the catalytic subunit of PRC2, contains a SET domain, which modifies transcription at the epigenetic level by affecting histone and DNA methylation [11]. There is evidence that EZH2 contributes to carcinogenesis by acting as an oncogene. Increased EZH2 levels are often correlated with a high grade of malignancy and poor prognosis. Stefansson [12] demonstrated that the EZH2-mediated epigenetic repression of DNA damage repair in breast tumors promoted the expansion of breast tumor-initiating cells, potentially contributing to cancer progression. The abrogation of EZH2 expression impairs the ability of colon cancer cells to move, resulting in anoikis, in a three-dimensional environment. Furthermore, EZH2 has been proposed as a potential metastasis marker [13]. Our group has previously studied the functions of EZH2 in cancer, demonstrating that aberrant upregulation of EZH2 was associated with vascular invasion and multidrug-resistance of liver cancer [14, 33]. Using miRNA target prediction algorithms, we identified many cancer-related genes, among which, EZH2 may be a potential direct target of miR-506. Therefore, EZH2 was chosen as the target gene for experimental analysis.

In the present study, we investigated the contribution of miR-506 to the proliferation and metastasis of colon cancer. Based on the findings that miRNA-506 expression was down-regulated in colon cancer tissues and was inversely associated with EZH2 expression, advanced clinical stage and lymph node metastasis, *in vitro* and *in vivo* experiments confirmed that miR-506 overexpression significantly inhibited cell proliferation and metastasis and that EZH2 is a direct target of miR-506. We also examined the effect of the miR-506-EZH2 axis on specific predicted downstream genes related to proliferation and metastasis and found that miR-506-EZH2 suppresses cell proliferation and metastasis by modulating multiple critical genes and the Wnt/β-catenin signaling pathway.

## RESULTS

### miR-506 expression was down-regulated in colon cancer tissues and inversely associated with EZH2 expression, advanced clinical stage and lymph node metastasis

**Figure 1:**
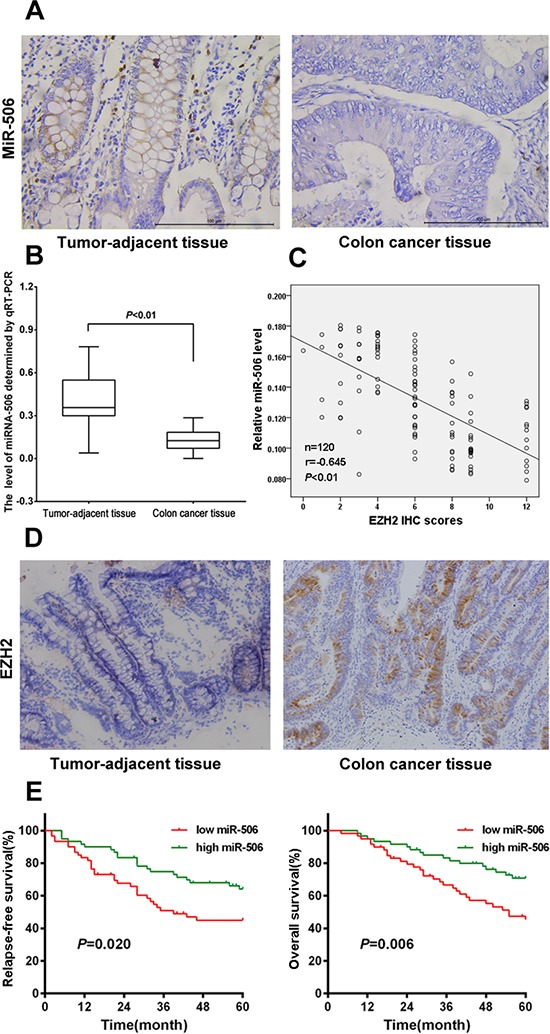
miRNA-506 expression was down-regulated and was inversely associated with EZH2 expression in colon cancer tissues, and high miR-506 expression predicted longer RFS and OS **A.** Analysis of miR-506 expression in colon cancer tissues and tumor-adjacent tissues by ISH (×400). **B.** The relative expression levels of miR-506 were assessed via qRT-PCR in 120 paired colon cancer and tumor-adjacent tissues. **C.** The expression of miR-506 was inversely correlated with the EZH2 immunohistochemical staining scores (*r* = −0.695, *p* < 0.01, Spearman's correlation analysis). **D.** EZH2 was overexpressed in the cytoplasm of colon cancer tissues but was nearly absent from the paired tumor-adjacent tissues (×200). **E.** Kaplan-Meier analysis revealed five-year OS rates of 45.27% and 70.80% and five-year RFS rates of 44.96% and 64.13% in the low and high miR-506 expression groups, respectively, suggesting that the OS and RFS rates of the low miR-506 expression group were higher than those of the high miR-506 expression group (*P* < 0.01).

To determine the expression of miR-506 in colon cancer tissues, mature miR-506 was detected in 120 paired colon cancer and tumor-adjacent tissues using qRT-PCR and ISH. The qRT-PCR results showed that the expression of miR-506 was significantly down-regulated in the colon cancer tissue compared to the adjacent tissue (*p* < 0.01, Figure 1B), and ISH analysis confirmed the expression pattern of miR-506 in tissues (Figure 1A), indicating that the down-regulation of miR-506 is a frequent event in colon cancer tissues.

We further analyzed the clinicopathological significance of miR-506 in colon cancer tissues. The relationship between the miR-506 expression levels and the clinicopathological characteristics of colon cancer patients are summarized in Table 1. The patients were stratified into 2 groups based on the median miR-506 expression levels; the miR-506 levels were negatively associated with tumor size (*p* = 0.027), lymph node invasion (*p* = 0.010), TNM stage (*p* = 0.034) and metastasis (*p* = 0.014).

**Table 1:** Relationship between miRNA-506 and clinicopathological parameters in 120 colon cancer patients

Variables	All cases	miRNA-506 expression		*χ*^2^	p*
		High (*n* = 60)	Low (*n* = 60)		
**Age (years)**					
≥60	88	42	46		
<60	32	18	14	0.289	0.409
**Gendar**					
Male	81	36	45		
Female	39	24	15	0.010	0.079
**Tumor size (cm)**					
<5	35	23	12		
≥5	85	37	48	0.001	0.027
**Depth of invasion**^a^					
m/sm/mp	49	29	20		
ss/se/si	71	31	40	0.014	0.095
**Tumor differentiation**					
Well	21	12	9		
Moderate	30	18	12		
Poor and others	69	30	39	0.565	0.246
**Lymph node invasion**					
Absent	37	25	12		
Present	83	35	48	0.000	0.010
**TNM stage**					
I–II	41	26	15		
III–IV	79	34	45	0.002	0.034
**Metastasis**					
Yes	45	16	29		
No	75	44	31	0.000	0.014

a m, tumor invasion of mucosa; sm, submocosa; mp, muscularis propria; ss, subserosa; se, serosa penetration; si, invasion to adjacent structures.

* *Probability, P, from χ^2^ test*.

We also examined the expression of EZH2 in 120 paired colon cancer tumor-adjacent tissues via IHC. High EZH2 expression (95/120) was detected in the cytoplasm of malignant cells whereas low EZH2 expression (84/120) was observed in the cells in the para-carcinoma tissue (Figure 1C). We further investigated the relationship of miR-506 expression with EZH2 expression. We found that the expression of miR-506 was inversely correlated with the intensity of EZH2 immunohistochemical staining (Figure 1B), suggesting that miR-506 negatively regulates EZH2 expression.

### Survival analysis according to miR-506 expression

We conducted a five-year follow-up of the patients. Kaplan-Meier analysis revealed that the five-year OS rate was 45.27% and 70.80% and that the five-year RFS rate was 44.96% and 64.13% in the low and high miR-506 expression groups, respectively, suggesting that the OS and RFS rates of the low miR-506 expression group were higher than those of the high miR-506 expression group (*p* < 0.01). Then, we further examined whether miR-506 is an independent prognostic factor based on multivariate Cox analysis. We found that the following factors were significantly related to survival: depth of invasion, lymph node invasion, TNM stage, metastasis and miR-506 expression level (Table 2). Our results indicated that miR-506 is not an independent prognostic factor.

**Table 2:** Multivariate survival analysis of five-year overall and relapse-free survival in 120 patients with colon cancer

variable	Overall survival			Relapse-free survival		
	Hazard Ratio	95% confidence interval	*P*	Hazard Ratio	95% confidence interval	*P*
**Age**	1.045	0.636–1.718	0.861	0.913	0.344–2.154	0.748
**Gendar**	1.339	0.528–2.395	0.538	1.288	0.783–2.117	0.319
**Tumor size**	2.246	0.900–5.605	0.083	2.333	0.974–5.589	0.057
**Depth of invasion**	3.415	1.330–8.772	0.011	3.513	1.428–8.643	0.006
**Tumor differentiation**	2.07	0.741–5.785	0.165	1.707	0.627–4.644	0.295
**Lymph node invasion**	1.962	1.140–3.374	0.015	2.025	1.181–3.472	0.010
**TNM stage**	1.525	1.010–2.543	0.036	2.013	1.117–3.749	0.026
**Metastasis**	3.533	2.181–5.774	0.000	2.901	1.815–4.830	0.000
**MiR-506**	0.328	0.171–0.632	0.001	0.444	0.238–0.830	0.011

### Correlation of miR-506 and EZH2 expression with the invasiveness of colon cancer cell lines

**Figure 2:**
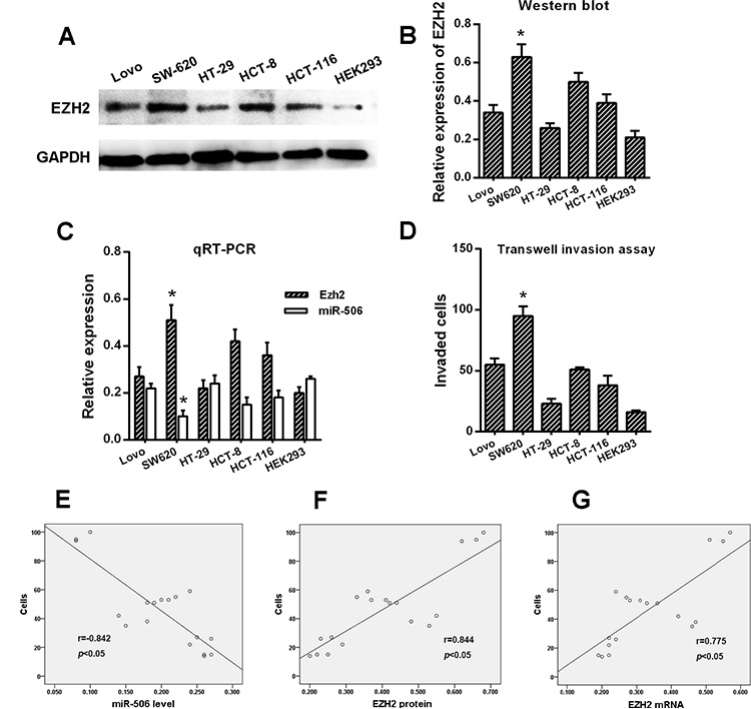
Correlation of miR-506 and EZH2 expression with the invasiveness of colon cancer cell lines **A–B.** The expression of EZH2 was examined via Western blot. EZH2 displayed significantly higher expression in SW620 cells than in the other cell types. Normal epithelial HEK293 cells were used as a control. **C.** The mRNA levels of miR-506 and EZH2 were assessed via qRT-PCR, and miR-506 displayed a significantly lower expression but displayed higher EZH2 expression in the SW620 cells compared to the other cell types. **D.** The SW620 cells exhibited significantly higher invasive capacity than the other cell types. **E–G.** The protein and mRNA levels of EZH2 were positively correlated with the invasiveness of colon cancer cells whereas the level of miR-506 was negatively correlated with the invasiveness of colon cancer cells. The data are shown as the means ± S.D. of three replicates (*p < 0.05).

To evaluate the correlation of miR-506 and EZH2 expression with the invasiveness of colon cancer, we detected their expression levels in colon cancer cells via qRT-PCR and Western blot. Normal epithelial cells, HEK293, were used as a control. The results showed that the miR-506 level in the SW620 cell line was significantly lower than in the Lovo, HT-29, HCT-8, HCT-116, and HEK293 cell lines (*p* < 0.05). However, the mRNA and protein levels of EZH2 in the SW620 cell line appeared to be higher than in the other five cell lines (*p* < 0.05, Figure 2A, 2B, 2C).

Our previous study [18] confirmed that the SW620 cell line exhibits higher invasive capacity than the other five cell lines based on a transwell assay (*p* < 0.05, Figure 2D). Considering that the SW620 cell line is more aggressive than the other cell lines, we performed the following experiment. Spearman's correlation was used to analyze the correlation between the levels of miR-506 and EZH2 in these six cell lines and the invasiveness of these six cell lines. The results suggested that the level of miR-506 negatively correlated with the invasiveness of colon cancer cells but that the expression of EZH2 positivity correlated with the invasiveness of colon cancer cells (*p* < 0.05, Figure 2E, 2F, 2G).

### The ectopic expression of miR-506 inhibited the proliferation, migration and invasion of colon cancer cells

**Figure 3:**
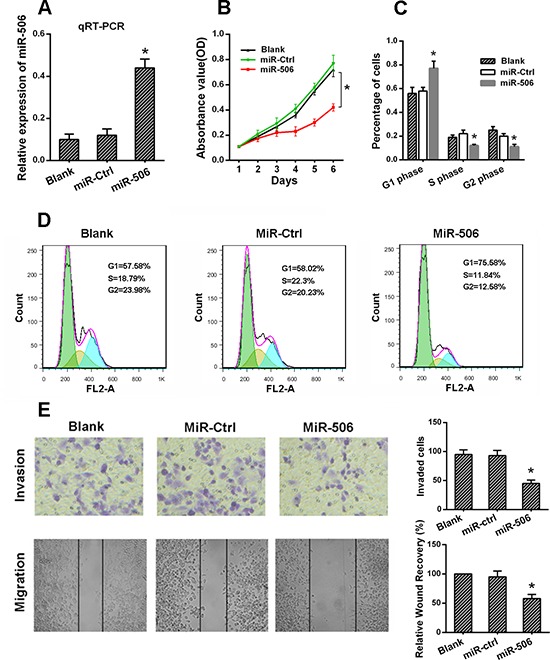
The ectopic expression of miR-506 inhibited the proliferation, migration and invasion of colon cancer cells **A.** miR-506 was up-regulated in SW620 cells via the transfection of a miR-506 mimic. After 48 h, the level of miR-506 was detected via qRT-PCR. **B.** The effect of miR-506 on the proliferation of SW620 cells was analyzed via an MTT assay. **C–D.** The effect of miR-506 on the cell cycle distribution of SW620 cells was monitored via flow cytometry. The miR-506-overexpressing SW620 cells were arrested at the G1 phase of the cell cycle, resulting in a corresponding reduction in the percentage of cells in the S and G2/M phases. **E.** Transwell and wound healing assays were utilized to analyze the effect of miR-506 on SW620 cell invasion and migration, respectively. The invasion and migration of SW620 cells were significantly reduced after the restoration of miR-506 expression. The data are shown as the means ± S.D. of three replicates (*p < 0.05).

To explore the potential role of miR-506 in regulating colon cancer progression, SW620 cells were infected with a miR-506 mimic to overexpress miR-506. The transfection efficiency was confirmed by qRT-PCR (Figure 3A). Then, we measured the effects of miR-506 overexpression on cell proliferation, migration and invasion. The MTT assay showed that miR-506 overexpression resulted in significant tumor growth inhibition in SW620 cells (Figure 3B). In contrast, SW620 cells transfected with a miR-506 inhibitor to knockdown miR-506 expression displayed increased growth (Figure S1). Cell cycle distribution analysis showed that the miR-506-overexpressing SW620 cells were arrested at the G1 phase, with a corresponding reduction in the percentage of cells in the S and G2 phases (Figure 3C, 3D). In addition, the migration and invasion capacities were evaluated via wound healing and transwell assays, respectively. The wound-healing assay demonstrated that the migration of miR-506-overexpressing SW620 cells into the wound was much slower than that of the miR-ctrl-transfected and blank cells. The invasiveness of SW620 cells, which was assessed using a transwell assay, was significantly reduced after the restoration of miR-506 expression (Figure 3E).

### miR-506 down-regulated EZH2 expression by directly targeting its 3′-UTR

**Figure 4:**
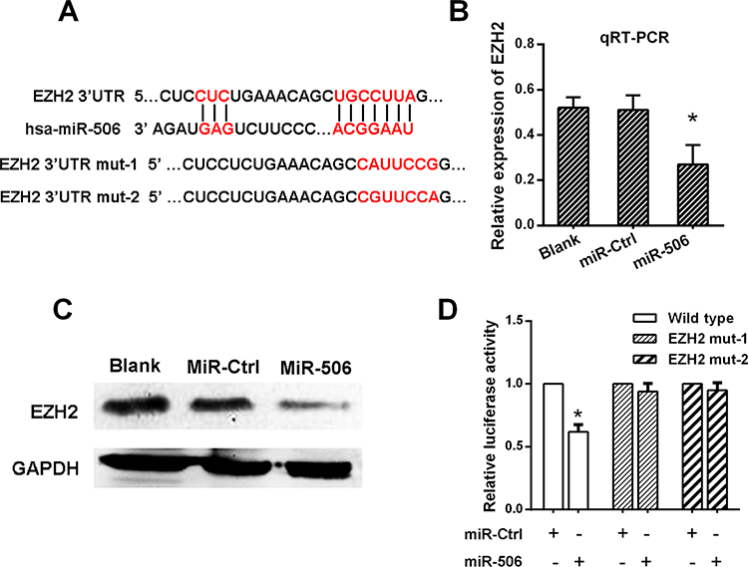
miR-506 down-regulated EZH2 expression by directly targeting its 3′-UTR **A.** The predicted sites of miR-506 binding to the 3′-UTR region of EZH2 were detected using bioinformatics prediction tools. The mutated site in the 3′-UTR region of EZH2 is shown. **B.** The mRNA level of EZH2 was decreased after transfection with the miR-506 mimic as demonstrated by qRT-PCR. **C.** The protein level of EZH2 was decreased after transfection with the miR-506 mimic as demonstrated by Western blot. **D.** The effect of miR-506 on the luciferase activity induced by the pMIR-EZH2-wt, pMIR-EZH2-mut1 and pMIR-EZH2-mut2 reporter plasmids in SW620 cells was measured via luciferase reporter gene assays. The data are shown as the means ± S.D. of three replicates (*p < 0.05).

To evaluate the biological mechanisms by which miR-506 promotes tumor growth and metastasis, we investigated the potential targets of miR-506 using target prediction programs (TargetScan, PicTar and miRanda). EZH2 was identified as a putative miR-506 target based on putative target sequences at position 36–42 of the EZH2 3′UTR (Figure 4A). Then, we analyzed EZH2 expression in SW620 cells via qRT-PCR and Western blot after transfecting these cells with the miR-506 mimic and the miR-506 inhibitor. As shown in Figure 4B, 4C and Figure S1, EZH2 expression was clearly reduced when miR-506 expression was restored and EZH2 expression increased when miR-506 was knocked down. To further verify this finding, we performed a luciferase reporter assay to determine whether EZH2 is a direct target of miR-506. The target region sequence of the EZH2 3′UTR (wild-type) or mutated sequence 1 (EZH2 mut-1) or 2 (EZH2 mut-2) was cloned into a luciferase reporter vector. These constructed reporter vectors were co-transfected with the miR-506 mimic or miR-ctrl into the SW620 cell line. The overexpression of miR-506 resulted in a significant decrease in the luciferase activity of the construct containing the wild-type 3′UTR of EZH2. This regulation was abolished when the nucleotides in the putative binding site were mutated (Figure 4D), indicating that the miR-506-mediated regulation of EZH2 expression depended on its binding to a specific seed region in the EZH2 3′UTR.

### The restoration of EZH2 expression reversed the proliferation and invasion of colon cancer cells

**Figure 5:**
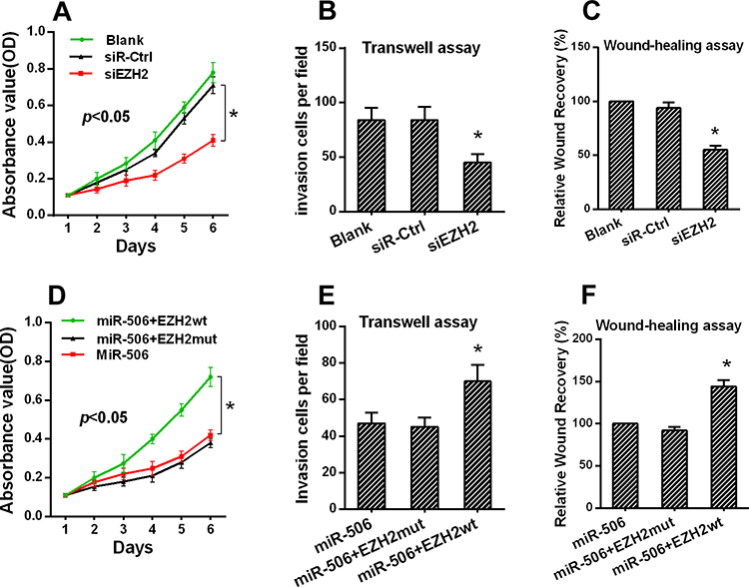
The restoration of EZH2 expression reversed the proliferation and invasion of colon cancer cells **A.** The proliferation of SW620 cells was inhibited after EZH2 silencing as demonstrated by an MTT assay. **B.** The invasion of SW620 cells was reduced as demonstrated by a transwell assay. **C.** The migration of SW620 cells was reduced after EZH2 knockdown. **D.** The proliferation of SW620 cells was reversed after co-transfection with the miR-506 and wild-type EZH2 plasmids as demonstrated by an MTT assay. **E.** The invasive capacity of SW620 cells was elevated following co-transfection with the miR-506 and wild-type EZH2 plasmids as demonstrated by a transwell assay. **F.** The migration capacity of SW620 cells was elevated following co-transfection with the miR-506 and wild-type EZH2 plasmids after EZH2 silencing. The data are shown as the means ± S.D. of three replicates (*p < 0.05).

Some studies have reported that silencing EZH2 expression inhibited the proliferation and invasion of colon cancer cells. Our findings supported these previous results. The MTT assays demonstrated that silencing EZH2 expression inhibited the proliferation of SW-620 cells (Figure 5A, Figure S2A, S2B). Furthermore, a Matrigel invasion assay and a wound-healing assay indicated that silencing EZH2 expression inhibited colon cancer cell invasion and migration, respectively (Figure 5B, 5C).

We hypothesized that the phenotypes associated with miR-506 expression would be reversed by the restoration of EZH2 expression. Therefore, we constructed an EZH2 expression plasmid and co-transfected this plasmid into SW-620 cells. The expression of EZH2 was confirmed by Western blot (Figure S2C, S2D). The MTT, Matrigel invasion and wound-healing assays demonstrated that the restoration of EZH2 expression significantly ameliorated the miR-506-induced suppression of colon cancer cell proliferation, invasion and migration, respectively (Figure 5D, 5E, 5F). Therefore, EZH2 plays an important role in the proliferation and metastasis of colon cancer cells, potentially by acting as a mediator of miR-506 function.

### miR-506-EZH2 activates/suppresses specific downstream tumor-associated genes and the Wnt/β-catenin signaling pathway

**Figure 6:**
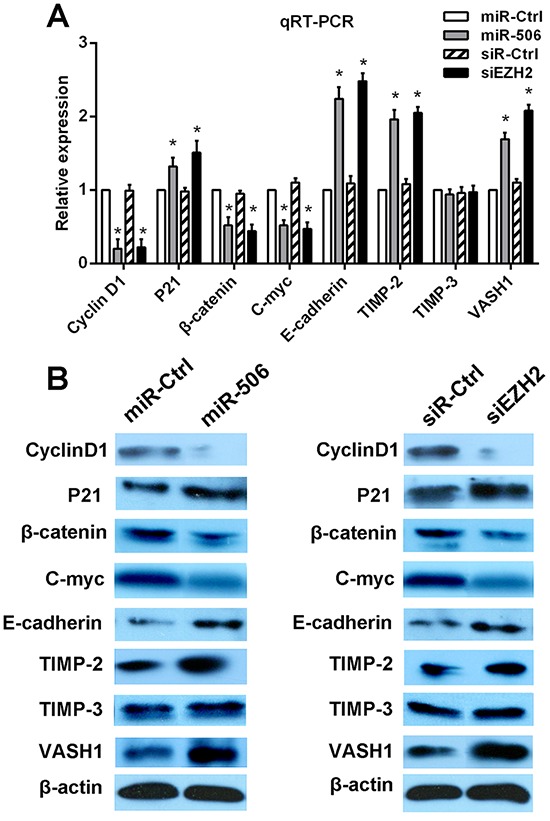
miR-506-EZH2 activates/suppresses specific downstream tumor-associated genes and the Wnt/β-catenin signaling pathway **A.** The mRNA expression levels of p21, cyclin D1, β-catenin, c-myc, TIMP-2, TIMP-3, E-cadherin and VASH1 were detected via qRT-PCR after transfection with the miR-506 mimic or EZH2 siRNA. **B.** The protein levels of these genes were analyzed via Western blot after transfection with the miR-506 mimic or EZH2 siRNA. The data are shown as the means ± S.D. of three replicates (*p < 0.05).

To further elucidate the mechanism by which the miR-506-EZH2 axis regulates the proliferation and metastasis of colon cancer, we measured the expression of specific downstream genes. First, we examined the G1/S phase proteins cyclin D1 and p21. Decreased cyclin D1 expression and increased p21 expression were detected after treatment with the miR-506 mimic or EZH2 siRNA (Figure 6). Then, we examined other tumor-associated genes, such as c-myc, E-cadherin, TIMP-2, TIMP-3, and VASH1. We found that treatment with the miR-506 mimic or EZH2 shRNA resulted in the up-regulation of the E-cadherin, TIMP-2, TIMP-3 and VASH1 genes and the down-regulation of the c-myc gene (Figure 6). β-catenin is a key downstream effector in the Wnt/β-catenin signaling pathway, which is associated with carcinogenesis and metastasis [19]. We confirmed that miR-506 overexpression or EZH2 silencing led to the down-regulation of β-catenin (Figure 6). Overall, these findings suggested that the miR-506-EZH2 axis suppresses tumor proliferation and metastasis by activating/suppressing the expression of specific downstream tumor-associated genes and the Wnt/β-catenin signaling pathway.

### miR-506 inhibits tumorigenicity and metastasis *in vivo*

**Figure 7:**
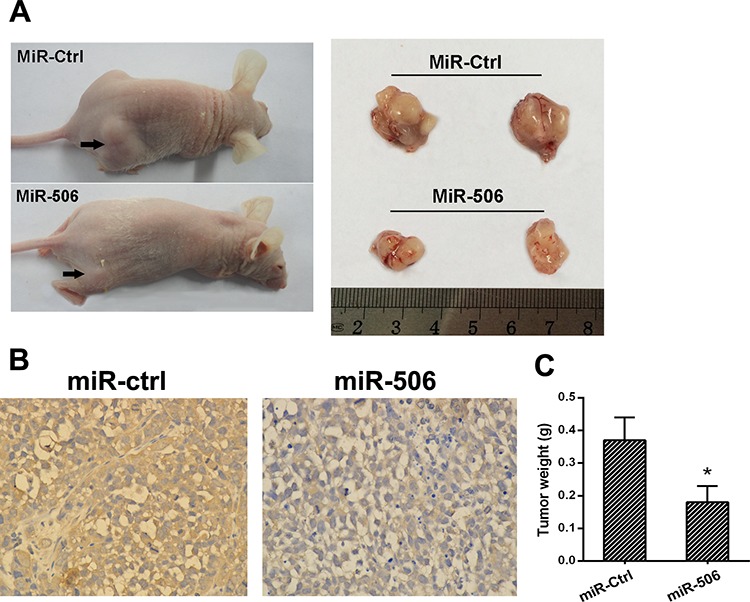
miR-506 inhibits tumorigenicity *in vivo* **A.** Tumors formed in nude mice. SW620 cells stably overexpressing miR-506 or empty vector were injected into the flanks of nude mice (*n* = 6), and the mice were sacrificed after 5 weeks. **B.** The histological images of resected tumors showed that the tumor tissue consisted of colon cancer cells. **C.** The weight of the tumors from the mice injected with miR-506-overexpressing SW620 cells was significantly less than that in those injected with miR-ctrl-expressing cells. The data are shown as the means ± S.D (*p < 0.05).

We then examined the role of miR-506 in tumorigenicity and metastatic potential *in vivo*. SW-620 cells stably overexpressing miR-506 were injected into the flanks of nude mice; cells stably expressing an empty vector were used as a control. After 5 weeks, the mice were sacrificed, and the tumors were collected. The results showed that the mice injected with miR-506-overexpressing SW620 cells exhibited a significantly smaller tumor size and tumor weight than those injected with the control cells (Figure 7A, 7C). The histological images of resected tumors showed that the tumor tissue consisted of colon cancer cells (Figure 7B).

**Figure 8:**
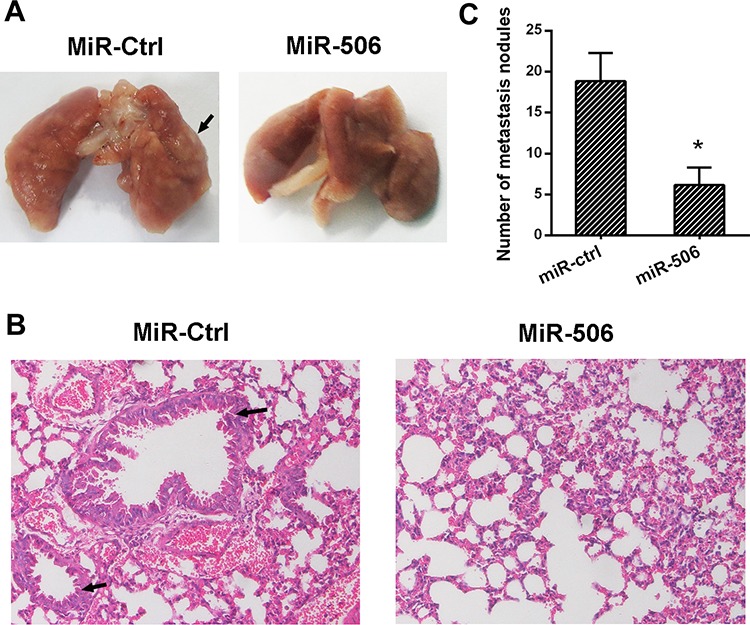
miR-506 inhibits tumor metastasis *in vivo* **A.** The lungs resected from the miR-506 and control groups. The lungs from the control group demonstrate swollen and obvious metastatic lesions (arrow) compared to the miR-506 group. **B.** Representative images of the histological assessment of the lungs via H&E staining (×200). The metastatic lesion is indicated by an arrow. **C.** The number of micro-metastatic nodules in the lung was significantly decreased in the mice injected with the lentiviral plasmid expressing miR-506. The data are shown as the means ± S.D (*p < 0.05).

To examine the metastasis of colon cancer cells, SW-620 cells stably overexpressing miR-506 or a mock control were intravenously injected into nude mice. At 6 weeks after this injection, the mice were sacrificed, the lungs were removed and the metastatic colonies in the lung were evaluated. The mice injected with miR-506-overexpressing SW620 cells exhibited a higher incidence of lung metastasis and more metastatic lesions than the mice injected with control cells (Figure 8A, 8B, 8C). These results suggested that miR-506 inhibits the tumorigenicity and metastatic potential of colon cancer cells *in vivo*.

## DISCUSSION

Emerging evidence has demonstrated that miRNAs play important roles in tumor development and progression. In our study, we found that miR-506 expression was down-regulated in colon cancer tissue and inversely correlated with EZH2 expression, tumor size, lymph node invasion, TNM stage and metastasis. Furthermore, *in vitro* and *in vivo* experiments confirmed that miR-506 inhibits the proliferation and metastasis of colon cancer cells. EZH2 was identified as a direct and functional target of miR-506 via the 3′UTR of EZH2. We also confirmed that the miR-506-EZH2 axis modulates proliferation and metastasis by regulating specific downstream tumor-associated genes and the Wnt/β-catenin signaling pathway.

To date, the role of miR-506 in cancer cells has not been well understood. miR-506 plays contradictory roles in different types of cancer. In melanoma, the overexpression of miR-506 was critical for maintaining cancer growth and invasion/migration [20], indicating that miR-506 acts as an oncogene in melanomas. In contrast, in ovarian cancer, miR-506 was identified as a key EMT inhibitor that suppressed cell migration and invasion, and miR-506 expression positively correlated with early FIGO stage and extended survival [21]. Similar results have been reported for liver cancer [22], cervical cancer [7] and breast cancer [23], indicating that miR-506 functions as a tumor suppressor gene in some tumors. The function of miR-506 in colon cancer was not well known. In our study, clinicopathological analysis revealed that miR-506 expression was inversely correlated with advanced clinical stage and lymph node metastasis. Based on *in vivo* and *in vitro* experiments, we confirmed that miR-506 acts as a tumor suppressor in colon cancer. We also investigated the correlation of miR-506 expression with the prognosis of colon cancer patients and found that the RFS and OS rates in the high miR-506 expression group were higher than those in the low miR-506 expression group, suggesting that elevated miR-506 expression may be a significant predictor of OS and RFS in colon cancer patients.

miRNAs typically perform their functions by suppressing the expression of target mRNAs. In ovarian cancer, miR-506 can suppress proliferation and induce senescence by directly targeting the CDK4/6-FOXM1 axis [24]. Further studies showed that miR-506 can inhibit cervical cancer growth by directly targeting the hedgehog pathway transcription factor Gli3 [7]. Moreover, recent profile studies showed that miR-506 regulated the biological behavior of cancer cells by targeting FLOT1 and GATA6 in renal cell cancer [25] and oral squamous cell cancer [26]. Our finding confirmed that miR-506 acts as a tumor suppressor in colon cancer, but the underlying mechanism is unclear. Therefore, the TargetScan, PicTar and miRanda databases were used to identify target genes of miR-506 in colon cancer. All three databases suggested that EZH2 may be a candidate miR-506 target gene, which is also a significant oncogene we are currently interested in studying. Then, we used a luciferase reporter assay to determine whether EZH2 is a direct target of miR-506. The results indicated that the regulation of EZH2 by miR-506 depended on its binding to the 3′UTR of EZH2. To establish whether the effect of miR-506 was exerted via the direct inhibition of EZH2, we restored EZH2 expression in miR-506-overexpressing cells and examined the proliferation and metastasis of these cells; we found that migration and proliferation were clearly increased, suggesting that EZH2 is a mediator of miR-506 function.

EZH2 is an oncogene that plays a critical role in cancer development by epigenetically silencing tumor suppressor gene expression [27]. Elevated expression of EZH2 has been described in a broad range of cancer types, such as prostate cancer [28], lung cancer [29], breast cancer [30], liver cancer [14] and colon cancer [31], and has been correlated with aggressive clinical manifestations. The results of our study were consistent with these previous reports.

The molecular mechanisms by which miR-506-EZH2 promotes cell proliferation and metastasis remain unknown. By reviewing published literature, we selected several cancer-related genes that may preliminarily explain the mechanism regarding how miR-506-EZH2 promotes cell proliferation and metastasis. We found that the miR-506-overexpressing colon cancer cells were arrested at the G1/S phase of the cell cycle. To further explore the underlying mechanism, the levels of the cell cycle proteins p21 and cyclin D1 were measured. p21 and cyclin D1 act as regulators of the G1/S transition [32, 33]. The genetic deletion of EZH2 results in the down-regulation of cyclin D1 in breast cancer [34] and in the up-regulation of p21 in ovarian cancer [35], causing cell cycle arrest at the G1/S phase. Our results were consistent with these findings, suggesting that miR-506-EZH2 promotes the proliferation of colon cancer cells by regulating p21 and cyclin D1. The Wnt/β-catenin signaling pathway is a vital signal transduction pathway, as the activation of this pathway contributes to carcinogenesis and cancer metastasis in certain types of cancer including colon cancer [36]. A recent study [37] revealed that EZH2 acts as a positive regulator of β-catenin, the key effector of the Wnt signaling pathway [19]. Another report confirmed that c-myc and cyclin D1 act as downstream genes of the Wnt/β-catenin pathway and that the transcription of c-myc and cyclin D1 is activated via the Wnt signaling pathway [34]. Our results showed that EZH2 silencing down-regulated β-catenin, c-myc and cyclin D1, suggesting that the miR-506-EZH2 axis may suppress tumor proliferation and metastasis by inhibiting the Wnt/β-catenin pathway. Moreover, to explore the mechanisms by which miR-506-EZH2 promotes metastasis, we examined several genes related to cell migration, such as E-cadherin, TIMP-2, TIMP-3 and VASH1. E-cadherin plays important roles in cell adhesion. siRNA-mediated EZH2 silencing in EC cells decreases their migration and invasion via the up-regulation of E-cadherin [38]. Tissue inhibitors of metalloproteinases (TIMPs) act as inhibitors of matrix metalloproteinases (MMPs). Shin et al [39] discovered that EZH2 plays an active role in this process by repressing the expression of TIMP-2 and TIMP-3 in prostate cancer cells. VASH1 is a negative regulator of angiogenesis. A recent study demonstrated that EZH2 silencing in tumor-associated endothelial cells inhibited angiogenesis via the reactivation of VASH1 [40]. In our experiments, we found that the down-regulation of EZH2 increased the mRNA and protein expression of E-cadherin, TIMP-2 and VASH1 but not TIMP-3. Taking together, these findings suggest that miR-506-EZH2 attenuates metastasis by modulating the expression of multiple migration-associated genes. In the present study, we found that up-regulating miR-506 led to the abrogation of EZH2 expression, which resulted in the inhibition of proliferation and metastasis of colon cancer cells, likely via the epigenetic modulation of specific downstream genes and the inhibition of the Wnt signaling pathway. However, hundreds of genes can be regulated by a single miRNA. EZH2 may not be the only gene targeted by miR-506, and other oncogenes targeted by miR-506 need to be explore in the future.

Based on the results of our study, we are the first to determine the biological function of miR-506 in colon cancer *in vitro* and *in vivo*. We found that miR-506 promotes the proliferation and metastasis of colon cancer. Further study demonstrated that EZH2 is a direct target gene of miR-506 that suppresses the growth and metastasis of colon cancer via the epigenetic modulation of specific downstream genes and the inhibition of the Wnt signaling pathway. Combined with the results of the previous studies mentioned above, this study not only complements the basic research of colon cancer but also provides a new putative marker to monitor and treat colon cancer. Clearly, some problems remain to be resolved; for example, the direct cause of the down-regulation of miR-506 in colon cancer is unclear. Epigenetic regulation, such as gene methylation or acetylation, may provide an explanation. This issue may become the focus and direction of our future research.

## MATERIALS AND METHODS

### Patient samples

This study was reviewed and approved by the ethics committee of Xiangya Hospital of Central South University, and written informed consent was obtained from all patients. The study included 120 patients with colon cancer aged from 39 to 77 years, all of whom underwent surgery from 2007 to 2009 at the Department of Gastrointestinal Surgery of Xiangya Hospital of Central South University. Clinicopathological characteristics, such as age, gender, tumor size, depth of invasion, tumor differentiation, lymph node invasion, TNM stage, and metastasis, were also examined. Tumors were classified and graded based on the pTNM classification advocated by the International Union against Cancer.

### Cell lines and cell culture

The colon cancer cell lines used in this study were purchased from American Type Culture Collection. These cell lines included Lovo, SW620, HT-29, HCT-8 and HCT-116. HEK293 cells were obtained from the Cancer Research Institute of Central South University, China. The HT-29 and HCT-8 cells were cultured in RPMI-1640 medium (Sigma-Aldrich Corp., St. Louis, MO, USA); the Lovo cells were cultured in F-12K medium (Sigma-Aldrich Corp.); the SW620 cells were cultured in L-15 medium (Sigma-Aldrich Corp.); the HCT-116 cells were cultured in McCoy's 5a medium (Sigma-Aldrich Corp.); and the HEK293 cells were cultured in modified Eagle's minimal essential medium (Sigma-Aldrich Corp.). All media were supplemented with 10% fetal bovine serum and 1% antibiotic/antimycotic solution (Biowest, Nuaille, France). All of the cell lines were cultured in 5% CO_2_ at 37°C in incubators at 100% humidity.

### Immunohistochemistry

Tissues were fixed in formalin and embedded in paraffin for immunohistochemistry according to previously published protocols [14]. Briefly, the tissue sections were de-paraffinized in xylene and rehydrated using a graded ethanol series. To quench endogenous peroxidase activity, the sections were immersed in 0.3% peroxidase-methanol solution for 30 minutes. For antigen retrieval, the sections were pretreated with citrate buffer for 15 minutes at 100°C in a microwave oven. The sections were hybridized with a primary antibody against EZH2 (Santa Cruz, USA) at 4°C overnight at a dilution of 1:100 and were visualized using the UltraVision Quanto Detection System HRP DAB kit (Thermo Scientific) according to the manufacturer's protocols. The stained sections were counterstained with hematoxylin, and photomicrographs were captured using an Olympus BX51 microscope. To investigate the number of EZH2-positive cells, we arbitrarily selected 10 high-power fields (×200) and counted the cancer cells under the microscope as described previously [14].

### *In situ* hybridization (ISH) analysis

ISH analysis was performed according to a previously described method [15]. Antisense oligonucleotide probes for miR-506 (Exiqon Inc., Woburn, MA, USA) were used for ISH.

### Western blot

Tissues or cells were homogenized and lysed with lysis buffer (50 mM Tris–HCl, 137 mM NaCl, 10% glycerol, 100 mM sodium orthovanadate, 1 mM phenylmethylsulfonyl fluoride (PMSF), 10 mg/ml aprotinin, 10 mg/ml leupeptin, 1% Nonidet P-40, and 5 mM protease inhibitor cocktail; pH 7.4). After the determination of the protein concentration using a BCA assay, β-mercaptoethanol and bromophenol blue were added to the sample buffer for electrophoresis. The proteins were separated via 10% PAGE and transferred to polyvinylidene difluoride membranes (Bio-Rad, USA). The membranes were incubated in a primary antibody overnight at 4°C. After incubation in a secondary antibody for 2 h, the reactive bands were visualized using an enhanced chemiluminescence system. The intensities of the bands were quantified using an image analysis system.

### Quantitative real time RT-PCR (qRT-PCR)

Total RNA was extracted from the cells or tissues using TRIzol (Invitrogen, Carlsbad, CA, USA) according to the manufacturer's protocol. For mature miR-506 detection, total RNA was polyadenylated using poly(A) polymerase (Ambion, Austin, TX, USA) as described previously [16]. Reverse transcription was performed using poly(A)-tailed total RNA, a reverse transcription primer and ImPro-II Reverse Transcriptase (Promega, Madison, WI, USA) according to the manufacturer's instructions. qRT-PCR was performed as described in the instructions provided with the Fast Start Universal SYBR Green Master Mix (Rox) (Roche Diagnostics GmbH Mannheim, Germany). The primers used for amplification were as follows: miR-506 forward 5′-TAAGGCACCCTTCTGAGTAGA-3′, reverse 5′-GCGAGCACAGAATTAATACGAC-3′; EZH2 forward (5′-GCCAGACTGGGAAGAAATCTG-3′), reverse (5′-TGTGCTGGAAAATCCAAGTCA-3′). GAPDH or β-actin was used as the internal control, other specific primers were purchased from Invitrogen Company.

### Cell cycle analysis

At 48 h after transfection, cells were harvested, washed with phosphate-buffered saline solution (PBS) and fixed in 70% ethanol at 4°C overnight. After fixation, the cells were washed twice with PBS before incubation in propidium iodide/RNase A solution (5 μg/ml propidium iodide and 100 mg/ml RNase A) at room temperature in the dark for 30 min. The stained cells were analyzed using a FACSCalibur flow cytometer (Becton-Dickinson, USA), and the analysis was completed within 30 min.

### MTT assay

At 24 h after transfection, cells were seeded on a 96-well plate at a density of 1 × 10^3^ cells/well. After incubation for 1, 2, 3, 4, 5 and 6 days at 37°C in a humidified incubator, 20 μl of MTT (5 mg/ml in PBS) was added to each well, and the cells were incubated for a further 4 h. After removal of the medium, 150 μl of DMSO was added to each well. The absorbance at a wavelength of 540 nm was recorded using a microplate reader.

### miRNA and siRNA transfection

The miRNA-506 mimic and the negative control were obtained from GenePharma (Shanghai, China). The sequence of the miR-506 mimic was 5′-UAAGGCACCCUUCUGAGUAGA-3′. Cells (5 × 10^5^ cells/2 ml/well) were seeded at 60% confluence in a six-well plate. After 48 h, the miRNA-506 mimic or the negative control was transfected into cells using Lipofectamine 2000 (Invitrogen, USA) at a final concentration of 50 nM according to the manufacturer's instructions. The siRNA targeting EZH2, with a sequence of 5′-AAGACTCTGAATGCAGTTGCT-3′, and the miR-506 inhibitor was purchased from GenePharma. The transfection of siRNA and miR-506 inhibitor was performed as described above. The final siRNA and miR-506 inhibitor concentration were 100 nM and 50 nM.

### Cell migration and invasion assays

The wound healing and transwell invasion assays were performed as described previously [17]. In brief, cells were seeded on six-well plates, incubated in their respective complete culture medium and grown to confluence overnight. The cells were scratched using a standard 200 μL tip. The debris was removed by washing the cells with serum-free medium. Serial photographs were obtained at different time points using a phase contrast microscope. The cell invasion assay was performed using Matrigel-coated transwell chambers (8-μm pore size, BD Biosciences, USA). Cancer cells were seeded above the Matrigel matrix in the upper chamber, and the bottom chamber was filled with culture medium containing a chemoattractant. The cells that permeated the Matrigel-coated membrane after 24 h were fixed with paraformaldehyde and then stained with crystal violet.

### Deletion of the miR-506 binding site on EZH2 and the luciferase reporter assay

The 3′-UTR of EZH2, which contained the predicted binding site of miR-506, was amplified from normal fetal genomic DNA via PCR using specific primers (EZH2– 3′-UTR forward 5′-catggcatactagtcatctgctacctcc-3′, reverse 5′-cctagcgcataagcttaaaacactttgc-3′). The PCR product was restricted and inserted between the restriction sites SpeI and HindIII into pMIR-REPORT-basic vector (Applied Biosystems, USA). The consensus miR-506 binding site was mutated via PCR using a QuikChange II XL site-directed mutagenesis kit (Stratagene, USA). All clones were verified by DNA sequencing. For the luciferase reporter assay, cells were seeded on 24-well plates. Then, 20 nM miRNA mimic or negative control was co-transfected with 100 ng of luciferase reporter vectors into SW620 cells using Lipofectamine 2000. At 48 hours after transfection, the cells were washed with Dulbecco's PBS (DPBS) and resuspended in lysis buffer, followed by the detection of luciferase activity using a luminescence reporter gene assay system (PerkinElmer, Norwalk, CT, USA) according to the manufacturer's instructions.

### Plasmid and lentivirus transduction

For EZH2 overexpression, a EZH2 cDNA sequence containing the putative miR-506 binding sites was cloned into the multiple cloning site of the pcDNA3.1 vector (Invitrogen, Carlsbad, CA, USA), which was termed wild-type 3′-UTR-EZH2 (wt 3′-UTR-EZH2). Additionally, mut 3′-UTR-EZH2 was generated as a control. In this experiment, cells cultured in a six-well plate were co-transfected with 50 nM miRNA mimic and 500 ng of a plasmid.

Lentiviral pEZX-MR04 plasmids expressing miR-506 or negative control miRNA were purchased from GeneCopoeia. The lentivirus expressing miR-506 or negative control miRNA was co-transfected into HEK293Ta cells using EndoFectin Lenti transfection reagent according to the manufacturer's instructions. After culturing for 48 hours, the lentiviral particles in the supernatant were harvested and filtered via centrifugation at 500 g for 10 min. SW-620 cells were then transduced with the lentivirus expressing miR-506 or negative control miRNA. To select stably transduced cells, the cells were resuspended and cultured in the presence of puromycin (2 μg/ml) for 2 weeks; qRT-PCR was performed to determine the level of miR-506 expression.

### *In vivo* tumor growth and metastasis assay

Six-week-old male BALB/c nude mice were obtained (Shanghai Slac Laboratory Animal Co. Ltd., China) and bred under specific pathogen-free conditions. SW-620 cells stably overexpressing miR-506 or empty vector were subcutaneously injected into the flank region of the mice (6 mice/group). Over a period of 5 weeks, tumor formation in the mice was observed by measuring the tumor volume. Then, the tumors were excised and weighed.

The tumor metastatic ability of the SW-620 cells stably overexpressing miR-506 or empty vector was determined by tail vein injection of the cells into 6-week-old male nude mice. After 6 weeks, the animals were ether-anesthetized, and their lungs were removed to determine the pulmonary metastatic foci. All animal experiments were reviewed and approved by the Ethics Review Committee of Central South University.

### Statistical analysis

All values are expressed as the means ± standard deviation (SD). The significance of the differences was determined via one-way ANOVA or Student's *t*-test. The χ2 test was used to evaluate the relationship between expression and the clinicopathological characteristics. Spearman's correlation coefficient was used to calculate the correlations between two groups. Kaplan-Meier analysis was employed for survival analysis, and the differences in the survival probabilities were estimated using the log-rank test. A Cox proportional hazards model was used to determine the independent factors affecting survival. *p* < 0.05 was considered to be significant. Statistical analysis was performed using SPSS version 17.0 (SPSS, Inc., USA).

## SUPPLEMENTARY MATERIALS FIGURES


