# Supplementary material for: MicroRNA-506 suppresses tumor proliferation and metastasis in colon cancer by directly targeting the oncogene EZH2
Source: Oncotarget. 2015 Oct 3;6(32):32586–601. doi: 10.18632/oncotarget.5309 (PMC4741714; doi:10.18632/oncotarget.5309)
Supplement: Supplementary file 1 [file oncotarget-06-32586-s001.pdf]

SUPPLEMENTARY FIGURES

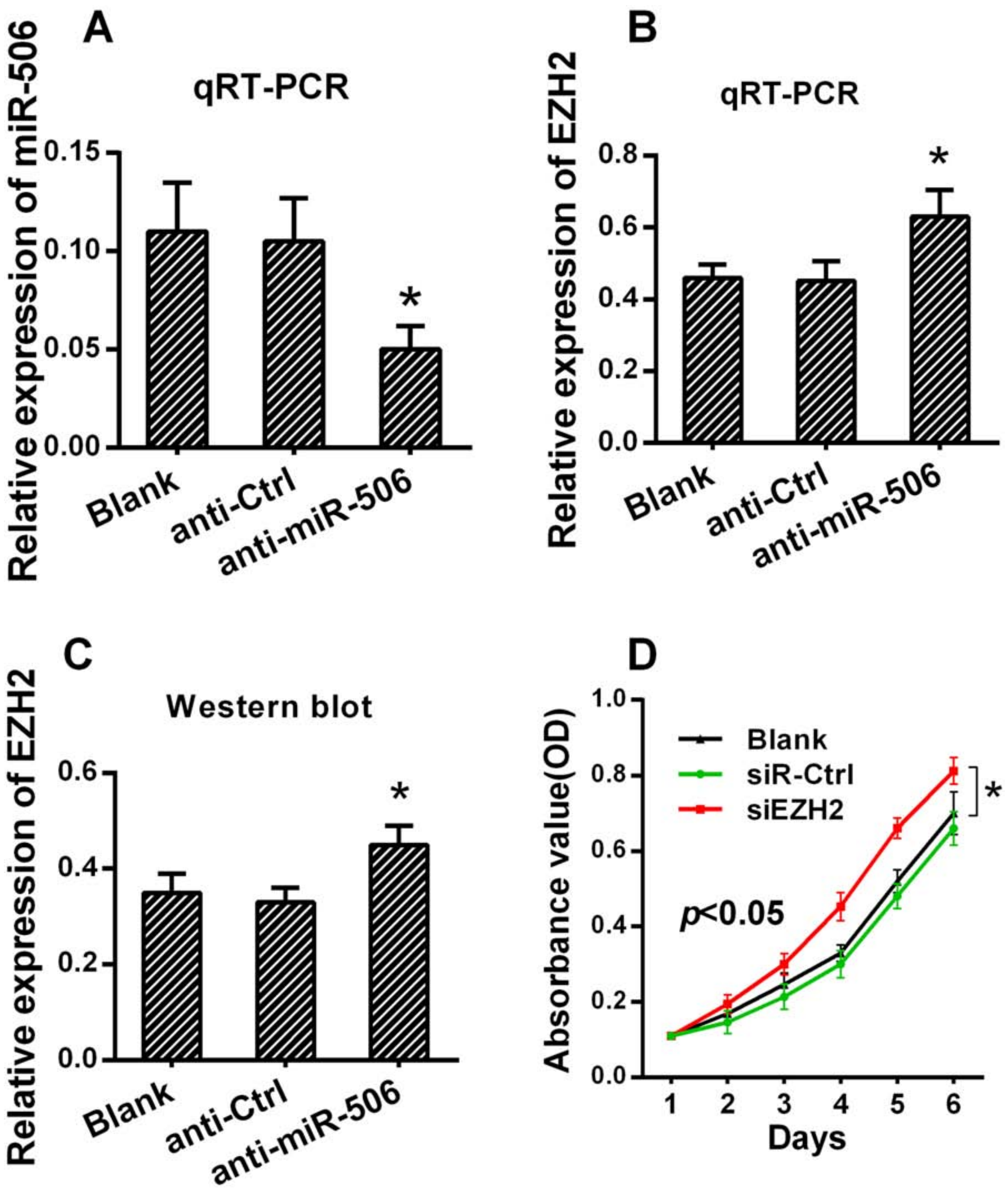

Supplementary Figure S1: miR-506 knockdown increases EZH2 expression and cell proliferation of colon cancer. A. The efficiency of miR-506 knockdown in SW620 cells. **B–C.** miR-506 knockdown increases EZH2 mRNA and protein levels in SW620 cells **D.** miR-506 knockdown promote the proliferation of SW620 cells. The data are shown as the means ± S.D (**p* < 0.05).

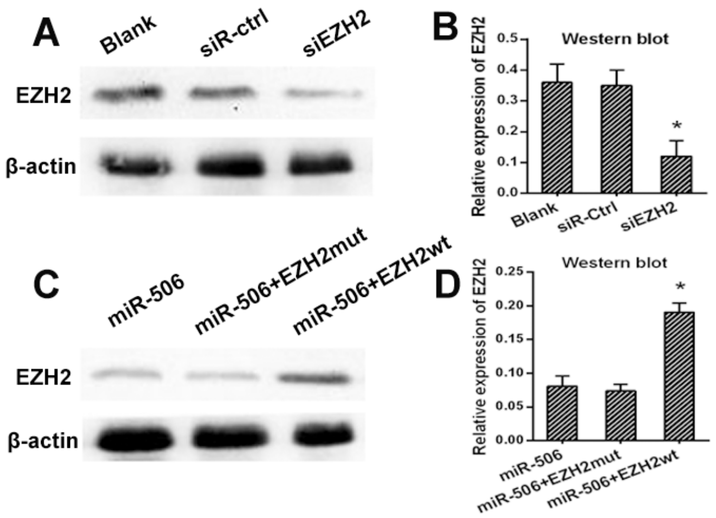

Supplementary Figure S2: EZH2 protein expression analyzed by Western blot. A–B. EZH2 expression was silenced using siRNA. The relative EZH2 expression level was normalized to β-actin expression. **C–D.** EZH2 expression was increased after co-transfection with the miR-506 and wild-type EZH2 plasmids compared to the other two treatments. The relative EZH2 expression level was normalized to β-actin expression. The data are shown as the means ± S.D. of three replicates (*$p < 0.05$).
